# The Clinical Characteristics and CT Findings of Parotid and Submandibular Gland Tumours

**DOI:** 10.1155/2021/8874100

**Published:** 2021-07-03

**Authors:** Houdong Zuo

**Affiliations:** Sichuan Key Laboratory of Medical Imaging, Department of Radiology, Affiliated Hospital of North Sichuan Medical College, Nanchong 637000, Sichuan, China

## Abstract

**Objective:**

To investigate the clinical characteristics and CT findings of parotid and submandibular gland tumours. *Materials and methods*. From May 2017 to April 2020, all patients with clinically proven parotid and submandibular gland enlargement and palpable masses underwent CT examinations. All patients were confirmed by pathology after surgery. The clinical characteristics and CT features were observed and evaluated. The mean density values before and after enhancement were measured and analyzed. The chi-square test, one-way ANOVA, and Student's *t*-test were used.

**Results:**

Ninety-four patients with a total of 94 unilateral tumours in the parotid and submandibular glands were enrolled, including 38 pleomorphic adenomas (PAs), 27 Warthin's tumours (WTs), and 29 malignant tumours (MTs). The majority of the PAs (28/38) and MTs (23/29) were located in the parotid gland; the others were located in the submandibular gland. All the WTs were in the parotid gland. The most common benign tumours of the parotid gland were PAs (28/38, 73.7%) and WTs (27/27, 100%), and the most common MTs were mucoepidermoid carcinoma, acinic cell carcinoma, and squamous cell carcinoma (4/29, 13.8%). The most common benign and malignant tumours in the submandibular gland were PAs (10/38, 26.3%) and ductal adenocarcinomas (3/4, 75%). The majority of PA patients (28/38) were female, compared with WT (2/27) (*P* < 0.001) and malignant tumour patients (10/29) (*P* < 0.01). A significant difference was also found between WTs and MTs in female patients (*P* < 0.05). The mean age of PA patients was 43.4 ± 12.1 years, which was lower than that of WTs (62.1 ± 11.7) and MTs (58 ± 14.18) (*P* < 0.001, *P* < 0.001, and *P*=0.244, respectively). On CT imaging, the mean diameter of the PAs and WTs was significantly smaller than that of the MTs (*P*=0.001 and *P* < 0.001), and no difference was observed between the PAs and WTs (*P*=0.275). In the parotid gland, the superficial lobe was more frequently involved than the deep lobe (PAs, 22 : 6; WTs, 17 : 10; and MTs, 15 : 8). The majority of PAs and WTs demonstrated round shapes (25/38, 19/27) and were well defined (30/38, 24/27); by contrast, most MTs were lobulated, irregular shapes (24/29), and ill defined (25/29). On plain CT, the PAs were usually homogeneous, while MTs were frequently heterogeneous, with more necrosis, larger cystic areas, and more haemorrhage or calcification. The mean CT values of PAs, WTs, and MTs were 39.2 ± 3.9 HU, 39.1 ± 3.0 HU, and 37.6 ± 3.1 HU (*P* > 0.05), respectively. On contrast CT, the WTs were significantly enhanced compared with MTs and PAs, with mean CT values of 53.5 ± 4.0 HU, 84.4 ± 6.0 HU, and 65.2 ± 3.8 HU, respectively (all *P* < 0.001). The mean CT value changes for PAs, WTs, and MTs (∆) were 14.4 ± 3.0 HU, 45.3 ± 4.5 HU, and 27.7 ± 2.5 HU, respectively. Significant differences were observed between ∆_PAs_ and ∆_WTs_, ∆_PAs_ and ∆_MTs_, and ∆_WTs_ and ∆_MTs_ (all *P* < 0.001).

**Conclusion:**

Parotid and submandibular gland tumours have some typical clinical characteristics and CT findings, and plain and early contrast-phase CT combined with clinical parameters may be helpful for diagnosis.

## 1. Introduction

Salivary glands are important exocrine organs in humans that can produce and release saliva and a variety of digestive enzymes into the oral cavity. Three pairs of large salivary glands—the parotid, submandibular, and sublingual glands—are distributed in the lining of the mouth. In 2017, the World Health Organization (WHO) classified salivary gland tumours into more than 30 malignant and benign histological subtypes and used cytology as a preliminary assessment tool rather than the original anatomical sites (WHO classification of tumours. Pathology and genetics of head and neck tumours. 4th ed. Lyon: IARC Press; 2017). Salivary gland neoplasms are relatively uncommon lesions of the head and neck, which constitute approximately 6% of all head and neck tumours [1, 2]. The morphology of salivary gland tumours is diverse due to their originating from different glandular cell types [3], which also demonstrate significant pathologic, biological, and clinical diversity. The current treatment strategies are surgical resection, radiation therapy (RT), chemotherapy, and multimodality therapy [3]. Surgical resection, subsequently followed with RT, is the standard therapeutic regimen. Chemotherapy has limited treatment effects and only brings some relief to patients with malignant and advanced disease. Additionally, patients with salivary tumours who undergo RT may experience some negative effects and harm, including xerostomia and salivary hypofunction due to damage caused to the salivary glands [4]. Furthermore, the therapeutic effect varies greatly depending on the tumour histology and stage [5].

The differentiation between benign and malignant tumours is crucial to the choice of treatment strategy and the quality of life and prognosis of patients because benign lesions can be treated by local excision, which has a lower complication rate and causes little harm. However, malignant tumours are the opposite, always requiring an expanded scope of resection and lymph node dissection, resulting in a more invasive experience for the patients [6, 7]. Imaging assessment plays an essential role in this setting, especially in identifying the location or nature of the tumour or even a correlation with the histology [8–12]. Computed tomography (CT) and magnetic resonance imaging (MRI) are the main imaging modalities used to evaluate tumours of the salivary glands, especially the parotid gland [13–15]. Although MRI has exhibited superior soft-tissue differentiation in salivary lesion evaluation, it also has some limitations, including contraindications for patients with internal ferromagnetic devices, high monetary and time costs, and the inability to identify and define stones and calcifications [16]. A variety of studies have been conducted on MRI findings to facilitate the differentiation of various types of parotid and submandibular gland tumours [15, 17–20], but few systematic CT analyses of common benign and malignant tumours of the salivary glands have been performed.

Therefore, this study aims to analyze the clinical imaging characteristics of CT findings in different types of parotid gland and submandibular gland tumours and investigate the role of CT in differentiating benign from malignant tumours with plain and early contrast-enhanced CT.

## 2. Materials and Methods

### 2.1. Patients

This retrospective study was approved by our institutional review board, and written informed consent was obtained from all patients.

From May 2017 to April 2020, all patients with clinically proven parotid and submandibular gland enlargement and a palpable mass underwent CT examination. All diagnoses were confirmed by pathology after surgery. The inclusion criteria were as follows: (1) CT examination performed in our hospital and including plain scan and contrast-enhancement scan; (2) good-quality CT images without artefacts; (3) masses that were not concomitant with other lesions, such as infections; and (4) masses diagnosed based on CT images. The exclusion criteria were as follows: (1) patients with absolute contraindications against CT and (2) patients with tumour recurrence after surgery. All patient data were extracted from clinical charts and the picture archiving and communication system (PACS) of our institution and processed on the attached workstation.

### 2.2. CT Protocol

All patients were examined with a 128-row multidetector CT scanner (Somatom Definition AS; Siemens Healthineers, Germany). Each scan was performed using 3 mm slice thickness, 120 kV, and 200 to 250 mA. A high-pressure automatic injector was used to inject 100 ml of nonionic iodinated contrast intravenously at a rate of 3 ml/s. The images were captured 30 seconds after completion of contrast injection. For each tumour, all tumour slices were evaluated, and then the mean density (CT values) of the lesion was calculated in Hounsfield units (HU) using a region of interest (ROI). The ROIs were drawn on the solid parts of the tumour, avoiding necrotic tissue, cystic degeneration, haemorrhage, and calcification. A standardized ROI was applied for all patients. The final diagnoses were determined by pathology after surgery. The changes in the CT values are marked with ∆: ∆_CT_ = (CT values)_plain_ − (CT values)_contrast_.

### 2.3. Statistical Analysis

All statistical analyses were performed with SPSS software (Version 19; IBM, New York, NY). Quantitative data are expressed as the means ± standard deviations. The chi-square test was used to analyze differences in categorical data, including gender and imaging characteristics. One-way ANOVA was used to analyze differences in ages. Student's *t*-test was used to calculate the differences of the tumour mean density between the plain and enhanced CT scans. *P* value < 0.05 was defined as statistically significant.

## 3. Results

### 3.1. Patient Demographic Data and Clinical Characteristics

Ninety-four patients (52 males and 42 females) with a mean age of 53.3 ± 15.2 (16–85 years) were included in this study. All patients underwent pathological confirmation and presented with unilateral masses or nodules for a total of 94 tumours in the parotid and submandibular glands (38 pleomorphic adenomas, 27 Warthin's tumours, and 29 malignant tumours). The malignant tumours included squamous cell carcinoma (6), mucoepidermoid carcinoma (4), acinic cell carcinoma (4), ductal adenocarcinoma (4), adenoid cystic carcinoma (3), secretory carcinoma (3), lymphoma (2), lymphoepithelioid carcinoma (1), myoepithelial carcinoma (1), and metastasis (1). The most common benign tumours of the parotid gland were pleomorphic adenomas (PAs) (28/38, 73.7%) and Warthin's tumours (WTs) (27/27, 100%). The most common malignant tumours (MTs) in the parotid gland were mucoepidermoid carcinomas (4/29, 13.8%), acinic cell carcinoma (4/29, 13.8%), and squamous cell carcinoma (4/29, 13.8%). The most common benign and malignant tumours in the submandibular gland were PAs (10/38, 26.3%) and ductal adenocarcinoma (3/4, 75%). Twenty-eight PAs, 27 WTs, and 23 MTs originated from the parotid gland, and 10 PAs and 6 MTs were in the submandibular gland.

Among the tumours, the majority of PA patients were female (28/38), while few females had WTs (2/27) (*P* < 0.001) or MTs (10/29) (*P* < 0.01). A difference was also found between WT and MT patients (*P* < 0.05).

The mean age of PA patients was 43.4 ± 12.1, which was lower than the WT (62.1 ± 11.7) and MT patients (58 ± 14.18). A difference was also observed between PA and WT patients (*P* < 0.001) and MT patients (*P* < 0.001), but no difference was observed between WT and MT patients (*P*=0.244) (Table 1).

### 3.2. CT Findings

The mean diameters of PAs in the short and long axis were 2.1 ± 0.8 cm (1.0 to 4.6 cm) and 2.3 ± 0.8 cm (1.3 to 5.0 cm), respectively. The mean diameters of WTs in the short and long axis were 1.9 ± 0.4 cm (0.8 to 2.5 cm) and 2.1 ± 0.5 cm (1.3 to 3.0 cm), respectively. The mean diameters of MTs in the short and long axis were 2.7 ± 1.4 cm (1.0 to 8.7 cm) and 3.4 ± 2.1 cm (1.3 to 11.8 cm), respectively. The mean diameter of the MTs was larger than that of the PAs and WTs (3.1 ± 1.8, 2.2 ± 0.8, and 2.0 ± 0.5 cm, respectively); a significant difference was observed between the PAs and MTs (*P*=0.001) and between the WTs and MTs (*P* < 0.001) but not between the PAs and WTs (*P*=0.275) (Table 1).

**Figure 1:**
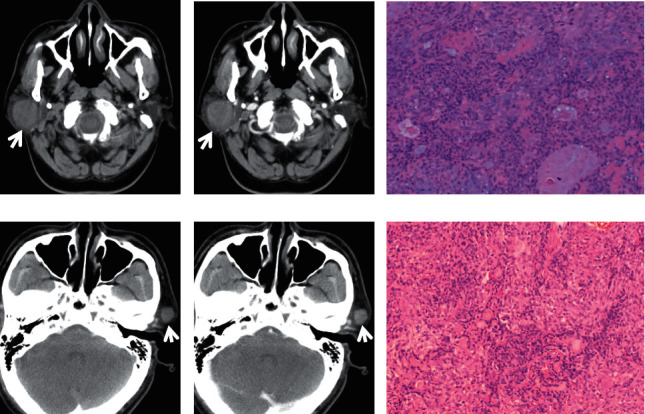
A 56-year-old male patient with PA in the right parotid gland. Plain CT showed that the PA was a well-defined, relative homogeneous, and round mass with 2.6 cm in diameter ((a), white arrow). The PA showed slight and inhomogeneous enhancement within 30 seconds after contrast injection ((b), white arrow). The PA was confirmed by pathology (HE staining) (c). A 52-year-old female patient diagnosed with PA in the left parotid gland. A well-defined, homogeneous, and round nodule with 1.4 cm in diameter was shown on plain CT ((d), white arrow). The nodule demonstrated moderate enhancement ((e), white arrow). HE staining (f).

**Figure 2:**
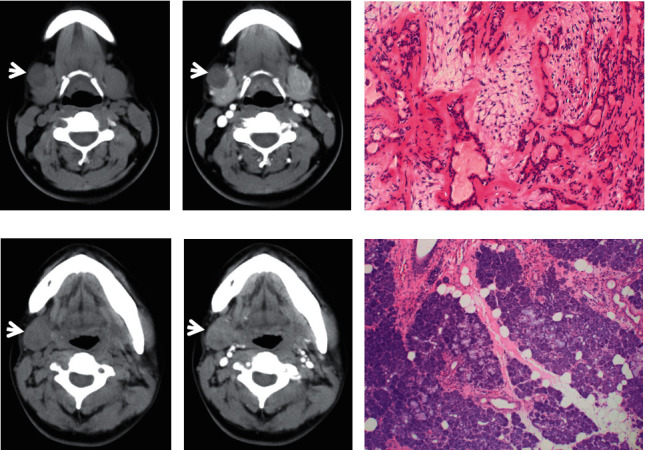
A 16-year-old female patient with PA in the right submandibular gland with a diameter of 1.9 cm. The lesion was a well-defined, homogeneous low-density, and round nodule ((a), white arrow). Slight enhancement was observed on contrast-enhanced CT ((b), white arrow). The PA was confirmed by HE staining (c). A 21-year-old female with PA in the right submandibular gland with a length of 2.6 cm in the long axis (d–f). The mass showed a well-defined, relatively homogeneous, slightly low-density, and round mass ((d), white arrow). Mild to moderate enhancement was observed on contrast-enhanced CT ((e), white arrow). The PA was confirmed by HE staining (f).

**Figure 3:**
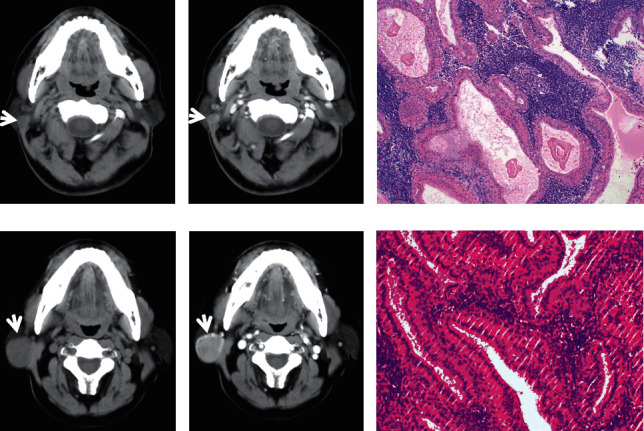
A 51-year-old male diagnosed with Warthin's tumour in the deep lobe of the right parotid with a length of 1.3 cm in the long axis. The tumour was a well-defined, homogeneous soft-tissue density, and round nodule ((a), white arrow). Enhancement of the tumour was obvious on contrast-enhanced CT ((b), white arrow). The WT was confirmed by HE staining (c). A 66-year-old male with WT determined by pathology in the right parotid with 2.8 cm in diameter. The tumour was a well-defined, homogeneous soft-tissue density, and round mass ((d), white arrow). Enhancement of the tumour was significant on contrast-enhanced CT ((e), white arrow). The WT was confirmed by HE staining (f).

**Figure 4:**
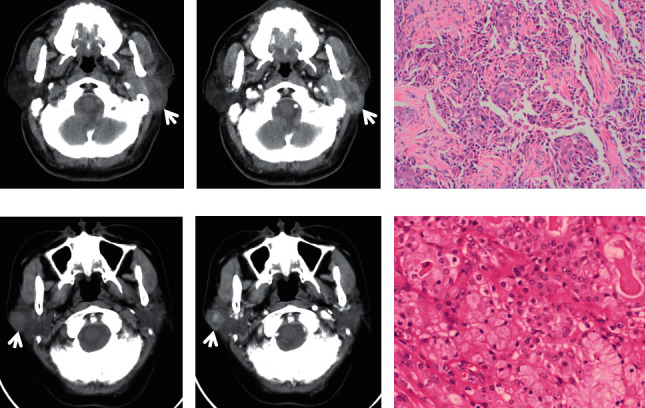
A 52-year-old male diagnosed with squamous cell carcinoma in the left parotid with a size of 5.4 cm in the long axis. The tumour was irregular, ill defined, and inhomogeneous on plain CT ((a), white arrow). Heterogeneous moderate enhancement was observed on contrast-enhanced CT (white arrow), and necrosis was observed inside the lesion (b). HE staining (c). A 51-year-old female presented with mucoepidermoid carcinoma in the superficial lobe of the right parotid. The tumour size was 2.0 cm in the long axis and was lobulated, ill defined, and inhomogeneous on plain CT ((d), white arrow). Heterogeneous moderate enhancement was observed on contrast-enhanced CT ((e), white arrow). HE staining (f).

**Figure 5:**
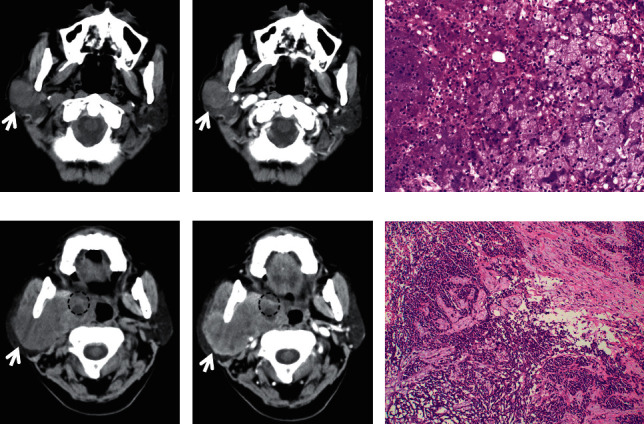
A 61-year-old female with acinic cell carcinoma in the right parotid. The tumour was 2.7 cm in diameter and presented as a lobulated, ill-defined, and inhomogeneous mass on plain CT ((a), white arrow). The tumour presented slight enhancement on contrast-enhanced CT ((b), white arrow). HE staining (c). A 67-year-old male with ductal adenocarcinoma in the right parotid gland. The tumour diameter was 7.9 cm in the long axis. The tumour was irregular, relative well defined, and inhomogeneous (d) and was enhanced moderately on contrast-enhanced CT ((e), white arrow). Necrosis could be seen on enhanced CT imaging (dotted-line circle). HE staining (f).

**Figure 6:**
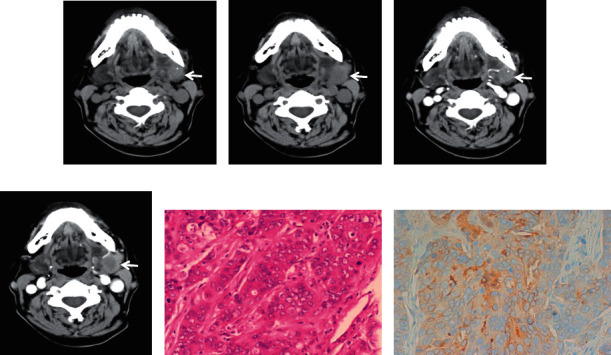
A 59-year-old male diagnosed with ductal adenocarcinoma in the left submandibular gland. A round, ill-defined, inhomogeneous nodule with little punctate calcification and 1.9 cm length in the long axis was shown on plain CT ((a, b), white arrows). The tumour demonstrated heterogeneous moderate enhancement, and a slightly low-density area could be seen (c, d). HE staining and immunohistochemistry confirmed the diagnosis of ductal adenocarcinoma (e, f). Immunohistochemistry: P63 (−), calponin (−), S-100 (−), CK7 (+), Ki-67 (+, 40%), GCDFP-15 (focal, +), and TTF-1 (−).

On CT imaging, most of the PAs (20/28), WTs (17/27), and MTs (15/23) were located in the superficial lobes in the parotid gland, and the others were located in the deep lobes. The majority of PAs and WTs demonstrated round lesions (25/38, 19/27), while the others showed lobulated (9/38, 6/27) and irregular shapes (4/38, 2/27). In contrast, a lobulated and irregular shape was observed in most MTs (24/29). A significant difference was observed between PAs and MTs and between WTs and MTs (all *P* < 0.001), but no difference was found between PAs and WTs (*P*=0.686). Most of the PAs and WTs were well defined (30/38, 24/27), whereas the majority of the MTs were ill defined (25/29). A significant difference was observed between PAs and MTs and between WTs and MTs (all *P* < 0.001), but no difference was found between PAs and WTs (*P*=0.288) (Table 1) (Figures 1–6).

The density of PAs was relatively homogeneous in 18 out of 38 patients, 16 patients showed heterogeneous density, cystic degeneration was observed in 4 patients, and no patients presented with calcification. In WTs, a uniform, slightly high density was observed in 18 patients, and a heterogeneous density with some cystic areas was observed in 9 patients; signs of calcification and haemorrhage were not observed on plain CT. The characteristics of MTs differed greatly, mainly demonstrating as solid masses with necrotic and cystic areas and haemorrhage. This was the greatest difference with PAs and WTs. The mean densities (CT value) of PAs, WTs, and MTs were 39.2 ± 3.9 HU, 39.1 ± 3.0 HU, and 37.6 ± 3.1 HU, respectively. No significant differences were observed between the mean density of PAs, WTs, and MTs (*P* > 0.05) (Table 1) (Figures 1–6).

On contrast CT images, the characteristics were different among PAs, WTs, and MTs on the early phase. The enhancement of WTs was most remarkable followed by that of MTs and PAs. The mean CT values of the PAs, WTs, and MTs were 53.5 ± 4.0 HU, 84.4 ± 6.0 HU, and 65.2 ± 3.8 HU, respectively. Significant differences were observed between PAs and WTs, PAs and MTs, and WTs and MTs (all *P* < 0.001). ∆_PAs_ was 14.4 ± 3.0 HU, ∆_WTs_ was 45.3 ± 4.5 HU, and ∆_MTs_ was 27.7 ± 2.5 HU. Significant differences were observed between ∆_PAs_ and ∆_WTs_, ∆_PAs_ and ∆_MTs_, and ∆_WTs_ and ∆_MTs_ (all *P* < 0.001) (Table 1) (Figures 1–6).

## 4. Discussion

In this study, we investigated the clinical characteristics and CT application in parotid and submandibular gland tumours to facilitate diagnosis with clinical and imaging data. We found that the most common benign tumours in the parotid and submandibular glands were pleomorphic adenomas (PAs) and Warthin's tumours (WTs), and the common malignant tumours were mucoepidermoid carcinomas, acinic cell carcinoma, squamous cell carcinoma, and ductal adenocarcinoma. The majority of PA patients were females, while most WT and MT patients were male. Patients with PAs were significantly younger than the WT and MT patients. On CT images, the mean diameter of the MTs was greater than 3 cm and larger than that of PAs and WTs, which were usually less than 3 cm in size. PAs and MTs located in the superficial lobe were more common than those located in the deep lobe of the parotid gland. The PAs and WTs were usually well-defined, round masses, and the MTs predominantly showed ill-defined, lobulated or irregular shapes. The PAs and WTs were homogeneous or heterogeneous with smaller cystic areas and little haemorrhage or calcification. However, MTs were usually heterogeneous with necrosis, cystic areas, haemorrhage, and little calcification inside. Additionally, the enhancement of WTs was much more obvious than that of PAs and MTs in the early phase of enhancement. The changes in density before and after contrast enhancement were of great help in differentiating benign and malignant tumours.

Salivary gland tumours (SGTs) are generic terms describing a large and diverse cohort of lesions characterized by morphologic heterogeneity. Some epidemiologic studies have investigated SGTs in different countries, with different results and divergences in the histologic classification and restrictions to specific populations, anatomical location, or tumour type [21–24]. Among the salivary glands, the parotid gland is the most frequently involved site followed by the submandibular gland, with a frequency of 42.3% to 70% in the parotid gland and 6.8%–11% in the submandibular gland [21, 22, 25]. Similar results were found in many large series on salivary gland tumours [23, 25, 26]. A similar result was found in the current study; we found that 83% of tumours were located in the parotid gland, and 17% were located in the submandibular gland, which is slightly higher than the previous report. The relatively small number of samples may account for this discrepancy. Additionally, among SGTs, the majority of tumours are benign, between 60% and 74.8% [21, 22]. Among the benign tumours, PAs are the most common, with an incidence up to 85.84% [21] followed by WTs, which are found in the parotid glands only. Similar findings were observed in most published series [21, 23, 24]. However, the most common malignant tumour is not the same in different investigations. Mucoepidermoid carcinomas are the most common malignancies according to Li et al. [21], Fonseca et al. [22], and Jansisyanont et al. [27] (18.75%, 31.4%, and 54.1%, respectively), but adenoid cystic carcinoma is the most common malignant tumour (33.7%) according to Lukšić et al. [24]. In this study, the most common malignant tumours were mucoepidermoid carcinomas, acinic cell carcinomas, squamous cell carcinomas, and ductal adenocarcinomas, which is similar with the previous studies.

Age is an important factor related to the incidence of SGTs. The age distribution in this study varied among patients with PAs, WTs, and MTs. The average age of PA patients was 43.4 ± 12.1, which was much smaller than that of WT patients (62.1 ± 11.7) and MT patients (58 ± 14.18), similar to some previous studies [21, 24, 28–30]. It has been reported that the average age of malignant tumour patients is higher than that of benign patients by approximately one decade [21, 22, 29]. However, Jansisyanont et al. [27] reported that patients suffering malignant tumours were younger than benign tumour patients by an average of 6 years.

According to some previous studies, female patients seem to be more affected than males [21, 23, 31, 32], but some studies reported an increased frequency in male patients [29, 33, 34]. However, the ratio of males to females is different for benign and malignant tumours. In a study by Fonseca et al. [22], the authors reported that the male-to-female ratio for benign tumours was 0.7 : 1, while malignant tumours demonstrated a ratio of 1.1 : 1, which suggested that females were commonly affected by benign tumours, but malignancies were more common in males. A clinical analysis by Li et al. [21] had a similar finding; in their report, the male-to-female ratio among all patients was 1.11 : 1, but the ratio was 0.99 : 1 for benign tumours and 1.34 : 1 for malignant tumours. In this study, the male-to-female ratio was 1.23 : 1 overall, 1.17 : 1 for benign tumour patients and 1.9 : 1 for malignancies, which was slightly different from the findings of some previous studies. Of note, among Warthin's tumour patients, there were only 2 females, leading to a high male-to-female ratio. However, in contrast to our and other previous studies, patients with malignancies were predominantly female in a study reported in Mexico [30].

Primary salivary gland tumours are uncommon in the head and neck, but it is very important to make an accurate preoperative diagnosis of the nature and learn the extent of the tumour because the surgeon needs to plan either a complete or a less extended surgery according to the tumour type and the incidence of recurrence, particularly among MTs and PAs or when accurate diagnosis of nature cannot be obtained in the deepest lobe of the glands. In this context, preoperative imaging evaluation is important for deciding clinical treatment and surgery options and can reveal not only the location, size, and number of masses in the same lobe or in the bilateral parotid gland but also the dynamic assessment of these tumours [35, 36]. Spiral CT is a valuable method for examining salivary tumours because of its high sensitivity and specificity, fast scanning, and high temporal and spatial resolution [16, 37]. CT showed an outstanding identification of not only the location and extension but also the nature of the tumour [38]. Therefore, CT was selected in this study.

The CT characteristics had a close correlation with the histopathology, especially the enhanced features. The contrast enhancement manifestations of salivary tumours are related to histopathologic traits and vascular architecture [39]. Therefore, the CT features were different from each other. In this study, the CT values were 39.2 HU, 39.1 HU, and 37.6 HU on plain CT scan, and no significant differences were observed. However, in the early contrast phase, the WTs enhanced significantly and rapidly, with a mean CT value of 84.4 ± 6.0 HU. By contrast, the enhancement of PAs and MTs was inferior to that of the WTs, with mean CT values of 53.5 ± 4.0 and 65.2 ± 3.8 HU, respectively. The difference in the enhancement may be due to the discrepancy in tumour histopathology and texture. It has been reported that the majority of WTs have a higher microvessel density and higher cellularity than PAs [39]. Woo et al. [40] found that the percentage of vessels per area in WTs was significantly higher than that in PAs (44.7% ± 6.3% in WTs and 15.4% ± 2.0% in PAs). Another explanation is that WTs have higher magnification, and a densely packed, capillary-like vessel network was shown in the papillary core of WTs; however, only a sparse collection of small arterioles was found in the PAs. Vascular endothelial growth factor receptor 2 (VEGF-R2) is well distributed in the epithelial cells of WTs and the blood vessel endothelium, unlike in PAs. This finding indicates that there is more active angiogenesis and a closer interaction with blood vessel endothelial cells in WTs than in PAs [40]. In general, these findings demonstrate that the contrast is delivered to WTs at a faster speed than PAs because of better perfusion through a denser network of blood vessels, resulting in rapid increased enhancement efficacy of WTs in the early CT phase.

MTs have common histopathologic features, and most MTs of the salivary glands have a high microvessel count and abundant stroma, but it may not be as mature as WTs [39], which may explain why MT enhancement was less significant than that of WTs but stronger than that of PAs.

Therefore, Warthin's tumours enhanced rapidly after contrast administration (within 30 seconds) and decreased during the late phase [38, 41, 42]. Similar results were also reported by Joo et al. [43] and Woo et al. [40], with average CT numbers of 94 ± 26 HU and 89 ± 21 HU, respectively. Unfortunately, the delay phase of contrast enhancement was not performed in this retrospective study.

It is reasonable to finish the CT examination of a patient with a suspected salivary mass as soon as possible, especially if the patient is uncooperative. CT scans with more than 2 phases would be helpful in displaying the characteristics of the tumour, but it is a time-consuming process that imposes a burden on patients in daily life. A quick and accurate diagnosis is beneficial to both radiologists and patients. The final confirmed diagnosis still depends on pathology, however. Therefore, in this study, we calculated the changes in CT values before and after enhancement, and we found that the CT value change of WTs was 45.3 ± 4.5, which was significantly higher than that of PAs (14.4 ± 3.0) and MTs (27.7 ± 2.5). Correct diagnoses are more likely after taking into account both clinical characteristics and CT features.

There are several limitations in this study. First, the number of samples was relatively small, especially for malignant tumours. Second, there were multiple malignant tumours in this study, and although the malignancies have some common characteristics, their heterogeneity is also more prominent than that of benign tumours, which may affect the results to some extent. Third, because this study was retrospective in nature, it lacked the delayed phase scan after contrast enhancement; therefore, the characteristics and comparisons with the early phase were not available. Fourth, fewer benign tumours were included; more convincing results could have been obtained by including more types of tumours. Fifth, MRI examinations were not performed in this study. Finally, we did not examine the relationship between the clinical and imaging parameters and pathology. Future studies will include improved results analyses.

In conclusion, our findings highlight the importance of clinical and CT characteristics in the evaluation of the parotid and submandibular glands, particularly in the diagnosis of WTs, PAs, and MTs. Female patients are frequently affected by PAs with relatively younger age, while WTs and MTs usually occur in the older males. PAs and WTs are generally less than 3 cm in diameter and usually present as well-defined, homogeneous, round masses, with little necrosis, less cystic areas, and little haemorrhage and calcification. By contrast, MTs are frequently more than 3 cm in diameter and demonstrate ill-defined, heterogeneous, lobulated, and irregularly shaped masses, with more necrosis, cystic areas, and haemorrhage or calcification. In the early enhanced phase of the CT scan, the WTs were significantly enhanced compared with MTs and PAs, which might be of great help for diagnosis and avoiding further delayed acquisitions and saving precious time for the doctors and patients.

## Figures and Tables

**Table 1:** Clinical characteristics and CT findings of PAs, WTs, and MTs.

	PAs (*N* = 38)	WTs (*N* = 27)	MTs (*N* = 29)	*P* value
Location				
Parotid	28 (73.7%)	27 (100%)	23 (79.3%)	
Superficial lobe	20 (71.4%)	17 (63%)	15 (65.2%)	
Deep lobe	8 (28.6%)	10 (37%)	8 (34.8%)	
Submandibular gland	10 (26.3%)	0 (0)	6 (20.7%)	
Gender				
Male	10 (26.3%)	25 (92.6%)	19 (65.5%)	*P*_*a*_ < 0.001; *P*_*b*_ < 0.01; *P*_*c*_ < 0.05
Female	28 (73.7%)	2 (7.4%)	10 (34.5%)	
Age	43.4 ± 12.1	62.1 ± 11.7	58 ± 14.18	*P*_*a*_ < 0.001; *P*_*b*_ < 0.001; *P*_*c*_=0.244
Mean diameter	2.2 ± 0.8 cm	2.0 ± 0.5 cm	3.1 ± 1.8 cm	*P*_*a*_=0.275; *P*_*b*_=0.001; *P*_*c*_ < 0.001
Short axis	2.1 ± 0.8 cm	1.9 ± 0.4 cm	2.7 ± 1.4 cm	
Long axis	2.3 ± 0.8 cm	2.1 ± 0.5 cm	3.4 ± 2.1 cm	
Shape				
Rounded	25 (65.8%)	19 (70.4%)	5 (17.2%)	*P*_*a*_=0.686; *P*_*b*_ < 0.001; *P*_*c*_ < 0.001
Lobulated/irregular	13 (34.2%)	8 (29.6%)	24 (82.8%)	
The boundary				
Well defined	30 (78.9%)	24 (88.9%)	4 (13.8%)	*P*_*a*_=0.288; *P*_*b*_ < 0.001; *P*_*c*_ < 0.001
Ill defined	8 (21.1%)	3 (11.1%)	25 (86.2%)	
Mean CT value (plain)	39.2 ± 3.9 HU	39.1 ± 3.0 HU	37.6 ± 3.1 HU	*P*_*a*_=0.759; *P*_*b*_=0.18; *P*_*c*_=0.09
Mean CT value (contrast)	53.5 ± 4.0 HU	84.4 ± 6.0 HU	65.2 ± 3.8 HU	*P*_*a*_ < 0.001; *P*_*b*_ < 0.001; *P*_*c*_ < 0.001
∆_CT_ values	14.4 ± 3.0 HU	45.3 ± 4.5 HU	27.7 ± 2.5 HU	*P*_*a*_ < 0.001; *P*_*b*_ < 0.001; *P*_*c*_ < 0.001

Note: *P*_*a*_ represents the *P* value between PAs and WTs, *P*_*b*_ represents the *P* value between PAs and MTs, and *P*_*c*_ represents the *P* value between WTs and MTs. Percentages are in parentheses.

## Data Availability

All the data in this study could be obtained upon request via e-mail to the corresponding author (zuohoud@163.com).
